# *ISG15* is downregulated by KLF12 and implicated in maintenance of cancer stem cell‐like features in cisplatin‐resistant ovarian cancer

**DOI:** 10.1111/jcmm.16503

**Published:** 2021-04-02

**Authors:** Qi Zhang, Jiamei Wang, Huaiyu Qiao, Lingyue Huyan, Baoqin Liu, Chao Li, Jingyi Jiang, Fuying Zhao, Huaqin Wang, Jing Yan

**Affiliations:** ^1^ Department of Biochemistry & Molecular Biology China Medical University Shenyang China; ^2^ Criminal Investigation Police University of China Shenyang China; ^3^ Clinical Medical Laboratory The 1st Affiliated Hospital China Medical University Shenyang China; ^4^ 5+3 Integrated Clinical Medicine 103K China Medical University Shenyang China

**Keywords:** cancer stem cell, cisplatin resistance, ISG15, KLF12, ovarian cancer

## Abstract

Drug resistance is often developed during clinical chemotherapy of ovarian cancers. The ubiquitin‐like protein interferon‐stimulated gene 15 (*ISG15*) is possibly dependent on tumour context to promote or suppress progression of various tumours. The ubiquitin‐like protein interferon‐stimulated gene 15 (*ISG15*) was decreased in cisplatin‐resistant ovarian cancer cells. The current study identified that both ectopic wild type and nonISGylatable mutant ISG15 expression inhibited CSC‐like phenotypes of cisplatin‐resistant ovarian cancer cells. Moreover, ectopic ISG15 expression suppressed tumour formation in nude mice. In addition, ISG15 downregulation promoted CSC‐like features of cisplatin‐sensitive ovarian cancer cells. Furthermore, low ISG15 expression was associated with poor prognosis in patients with ovarian cancer. Transcriptional repressor Krüppel‐like factor 12 (KLF12) downregulated ISG15 in cisplatin‐resistant cells. Our data indicated that downregulating *ISG15* expression, *via* weakening effect of KLF12, might be considered as new therapeutic strategy to inhibit CSC phenotypes in the treatment of cisplatin‐resistant ovarian cancer.

## 1 INTRODUCTION

High morbidity and mortality make ovarian cancer the most deadly gynaecologic cancer.^1^ Platinum‐based chemotherapy following optimal surgical excision of the tumour is currently considered as the standard therapy for ovarian cancer. Even though most ovarian cancers initially respond to chemotherapy with relative sensitivity, drug resistance is often developed during clinical chemotherapy, thereby leading to shorter overall survival and worse prognosis.^2^ Consequently, it is critical to develop effective strategies to improve cancer treatment outcomes.

Interferon‐stimulated Gene 15 (*ISG15*) is a type I interferon‐inducible gene and implicated in interferon‐induced immune responses.^3^ ISG15 is also known as ubiquitin cross‐reactive protein (UCRP), and its structure is very similar to ubiquitin.^4^, ^5^ Much like ubiquitination, a process called ISGylation can be undertaken by conjugation of ISG15 to a lysine residue in the target proteins by enzyme cascade reaction.^6^ Recently, it has been reported that ISG15 plays a strikingly ambiguous role in cancers. Free ISG15 and conjugated ones are increased and acted as important oncoproteins.^7^, ^8^, ^9^, ^10^, ^11^, ^12^ On the other hand, ISG15 suppresses tumour progression by regulating the production of IFN‐γ and the functions of natural killer cells, enhancing E3 ubiquitin ligase activity of the carboxyl terminus of Hsp70‐interacting protein^13^ and inhibiting cancer cell growth and promoting apoptosis.^14^ Furthermore, free form ISG15 can also be released extracellularly and alters the tumour microenvironment *via* functioning as an immunomodulatory cytokine.^7^, ^15^, ^16^ Recently, our group have also revealed ISG15 suppresses translation of ABCC2 *via* ISGylation of hnRNPA2B1 and enhances drug sensitivity in cisplatin‐resistant ovarian cancer cells.^17^

Krüppel‐like factors (KLFs) are homologues of the *Drosophila melanogaster* Krüppel protein, which manages body segmentation during *Drosophila* embryo developing.^18^ KLFs contain evolutionarily conserved zinc (Zn)‐finger domains in their C‐terminal regions. KLFs and specificity proteins (SPs) share high similarity with regards to their structure and DNA‐binding capacity, thereby they are commonly known as SP/KLF transcription factors.^19^ To date, 18 KLFs are reported in research work. These transcription factors participate in a variety of key cellular processes in normal tissues, including proliferation, differentiation, pluripotency and homeostasis.^20^, ^21^, ^22^ In recent studies, it is uncovered that KLFs expression and activity are altered in individual cancers,^23^, ^24^, ^25^, ^26^ even one certain KLF can function as tumour promoter and suppressor based on tumour types or stage.^26^, ^27^ KLF4 is extremely important for preserving CSC‐like properties in breast cancer,^23^ colorectal^28^ and pancreatic cancer cells.^29^ KLF4, especially, can induce cells with cancer stem cell properties through somatic reprogramming.^30^, ^31^ In both normal stem cells and CSCs, KLF4 can maintain telomerase activity, which reveals a role for KLF4 in long‐term proliferative potential of stem cells.^32^ Several other KLF family members have been involved in CSCs, but their regulatory functions and mechanisms are not clarified. For example, KLF5 encourages CSC viability in ovarian cancer,^24^ and KLF9 inhibits glioblastoma‐initiating stem cells.^25^

The current study displayed that ISG15 evidently decreased in cisplatin‐resistant cell lines, when compared with their cisplatin‐sensitive partners. Ectopic expression of wild‐type ISG15 elevated cellular responses to cisplatin as well as ectopically expressed both wild‐type ISG15 and nonISGylatable mutant ISG15 leaded to decreasing CSC population in resistant cell lines. Although *ISG15* knock‐down in parental cell lines maintained the sensitivity in cisplatin, attenuated ISG15 strengthened CSC features of the cells. In addition, it was shown in the present study that ISG15 expression was negatively regulated by KLF12. Therefore, these studies suggested that KLF12 might function as a potential therapeutic target *via* regulating *ISG15* expression for inhibition of CSC phenotype in the treatment of cisplatin‐resistant ovarian cancer.

## 2 MATERIALS AND METHODS

### 2.1 Cell line cultivation

SKOV3 and A2780 cells were acquired from ATCC and GenChem, respectively. The initial cells were identified using STR profiling. Initial dose‐response studies of cisplatin over 72 hours showed that IC50 of SKOV3 and A2780 cells were 0.72 ± 0.13 and 1.21 ± 0.23 μg/mL, respectively. Cisplatin‐resistant variants of each cell line (SKOV3/DDP and A2780/DDP) were generated by continuous exposure to cisplatin with IC50 concentrations for 72 hours and then allowed to recover for a further 72 hours. This procedure was carried out for approximately 6 months, and IC50 of SKOV3/DDP and A2780/DDP cells was reassessed to be 5.72 ± 0.38 and 11.23 ± 0.46 μg/mL, respectively. Cells were then maintained continuously in the presence of cisplatin with these new IC50 concentrations for a further 6 months. Two pairs of cisplatin‐sensitive (SKOV3 and A2780) and cisplatin‐resistant (SKOV3/DDP and A2780/DDP) human ovarian cancer cell lines were cultured in RPMI1640 (Life Technologies) containing 10% foetal bovine serum (FBS, Sigma), 100 IU/mL of penicillin and 100 µg/mL of streptomycin (Sigma).

### 2.2 Cytotoxicity assay

6 × 10^3^ cells/well were cultured in 96‐well plate for 24‐hour attachment and then incubated with 10 μg/mL of cisplatin for additional 48 hours. Cell viability was analysed using Cell Counting Kit 8 (CCK‐8) (Dojindo Laboratories).

### 2.3 Apoptosis assay

Flow cytometry was performed following PI‐ and FITC‐labelled annexin V staining according to the manufacturer's protocol (KeyGen Biotech, Nanjing, China). Briefly, after 48‐hour incubation, cells were washed, resuspended in 200 μL binding buffer at 1 × 10^6^ cells/mL and incubated with 5 μL FITC‐annexin V. After 15‐minute incubation at room temperature in the dark, 300 μL binding buffer together with 5 μL PI was added to each tube. The samples were incubated for 30 minutes at room temperature in the dark. Flow cytometry was performed within 1 hour.

### 2.4 Transwell migration and invasion assays

In vitro transwell migration assays were performed in modified Boyden chambers with 8‐mm‐pore filter inserts in 24‐well plates (BD Biosciences, San Jose, CA, USA). Briefly, the lower chamber was filled with DMEM containing 10% foetal bovine serum. A2780 and SKOV3 cells were collected after trypsinization, resuspended in 200 mL of conditional medium collected and transferred to the upper chamber. After 24 hours of incubation, the filter was gently removed from the chamber, and the cells on the upper surface were removed using a cotton swab. Cells fixed with 4% paraformaldehyde for 15 minutes and stained with 0.1% crystal violet.

In transwell invasion assay, the cells went through the Matrigel matrix membrane of the upper chamber. After 24 hours of incubation, the filter was gently removed from the chamber, and the cells on the upper surface were removed. Then cells were fixed and stained.

### 2.5 Spheroid forming assay

10^4^ cells/well were seeded in ultra‐low‐attachment six‐well plates (Corning, Acton, MA, USA) and cultured in serum‐free DMEM/F12 containing B27 (1:50, Invitrogen, Carlsbad, CA, USA), 20 ng/mL human recombinant EGF (epidermal growth factor, Sigma‐Aldrich, Saint Louis, MO, USA), 20 ng/mL bFGF (basic fibroblast growth factor, Sigma‐Aldrich), 4 μg/mL heparin (Sigma‐Aldrich) and 5 μg/mL insulin (Sigma‐Aldrich). Spheroids were imaged and its numbers were counted under phase‐contrast microscopy after 7‐10 days of cell seeding. Only the spheroid exceeding 50 μm in diameter was counted in the result.

### 2.6 Luciferase reporter assay

The DNA fragments −1767/+53, −1237/+53, −896/+53 and −411/+53 of human *ISG15* promoter were inserted into the upstream of a firefly luciferase gene in luciferase reporter vector. As well as, luciferase reporters bearing ISG15 promoter with −1130/−1126 sequence deletion, or −598/−594 sequence deletion, or both of them deletion were constructed. Cells were cotransfected with the indicated luciferase reporter and Renilla luciferase (pRL‐TK) as a normalizing control. 48 hours later, luciferase activity was measured using Dual‐Luciferase Reporter Assay (Promega) according to the manufacturer's protocols. Transfections were performed in triplicates, and three experiments were repeated independently.

### 2.7 Lentiviral vector construction and recombinant lentivirus production

Gene encoding ISG15 and ISG15 (G156/157A) was cloned into the lentiviral vector (GeneChem Co., Ltd., Shanghai, China). DNA sequencing was performed by GeneChem to verify the sequence of the insert, and the identities were 100%. Following construction, A2780 and SKOV3 cells were cotransfected. Recombinant lentiviruses were harvested at 48 and 72 hours post‐transfection, centrifuged to get rid of cell debris and then filtered. Ultimately, a concentrated lentivirus solution was obtained.

### 2.8 Western blot analysis

Cells were homogenized in lysis buffer (20 mmol/L Tris‐HCl, 150 mmol/L NaCl, 2 mmol/L EDTA, 1% Triton‐X100 ) containing freshly added protease inhibitor cocktail (Sigma‐Aldrich). Quantification of extracted proteins was performed using the BCA protein assay kit. 20 μg of total protein was subjected to 12% SDS‐PAGE and then transferred to PVDF membrane (Millipore Corporation). Antibody of ISG15, ISG15‐Flag and KLF12 was diluted at 1:1000. And GAPGH antibody was diluted at 1:5000.

### 2.9 Dot blot

3 × 10^5^ cells/well were seeded into six‐well plates with RPMI1640 containing 10% FBS, and the culture medium was replaced by the serum‐free RPMI1640 after cell attachment. The cells were incubated for additional 3 days, and the supernatant was collected and centrifuged. Dot blot was performed by loading 100 μL of the supernatant on the NC membrane. The blots on the NC membrane were blocked with 5% skim milk for 1 hour and reacted with specific primary ISG15 antibody. ISG15 antibody was diluted at 1:1000. The acquired signals were detected using the ECL Western blotting system.

### 2.10 Real‐time reverse transcription polymerase chain reaction (RT‐PCR)

Total RNAs were isolated using TRIzol reagent (Invitrogen) and reversely transcribed using SuperScript™ II RNase H‐reverse transcriptase (Invitrogen). Amplification of cDNA was performed using SYBR Green PCR Master Mix (Applied Biosystems) on LightCycler480 II System (Roche). Each data was normalized against *GAPDH* and presented as ratio vs vehicle‐treated control. The experiments were repeated for three times in triplicate.

### 2.11 Label and capture nascent RNA

Labelling and isolation of newly synthesized RNA were performed using the ClickiT Nascent RNA Capture kit (Thermo Fisher Scientific) as previously reported.^33^ Briefly, after pulsing with 0.2 mmol/L 5‐ethynyl uridine (EU) for 4 hours, total RNA was isolated and subjected to nascent RNA capture, followed by analysis using real‐time PCR.

### 2.12 Analysis of mRNA stability

5 μg/mL of actinomycin D is selected as the optimal concentration to inhibit ISG15 transcription. Cells were exposed to 5 μg/mL of actinomycin D for the indicated time, and total RNA was isolated and analysed by quantitative RT‐PCR. ISG15 mRNA expression was normalized to 18S rRNA. The value at time zero was set at 100%, and data were presented as a percentage of the value at time zero from three experiments repeated independently.

### 2.13 Chromatin immunoprecipitation (ChIP)

Chromatin immunoprecipitation assay was performed using the Upstate Biotechnology Inc kit, and the detailed protocol was as previously reported.^34^ The output DNA produced using this protocol was analysed using qPCR.

### 2.14 Nude mice xenograft experiments

BALB/c‐nu/nu mice (4‐5 weeks old, female) (Beijing Vital River Laboratory Animal Technology) were subcutaneously inoculated with the serially diluted viable SKOV3/DDP cells. Euthanasia of the experiment mice were performed using overdose of sodium pentobarbital on day 28, and primary tumours were excised and weighed. All animal procedures were approved and compiled with the guidelines of the Institutional Animal Care Committee of China Medical University.

### 2.15 Statistics

ANOVA and post hoc Dunnett's test were used to analyse the statistical significance of the difference, which was defined as *P* < .05. All experiments were repeated three times independently, and data from a representative experiment were presented as the mean ± SD (standard deviation).

## 3 RESULTS

### 3.1 ISG15 suppresses cancer stem cell‐like features in ovarian cancer cells

**FIGURE 1:**
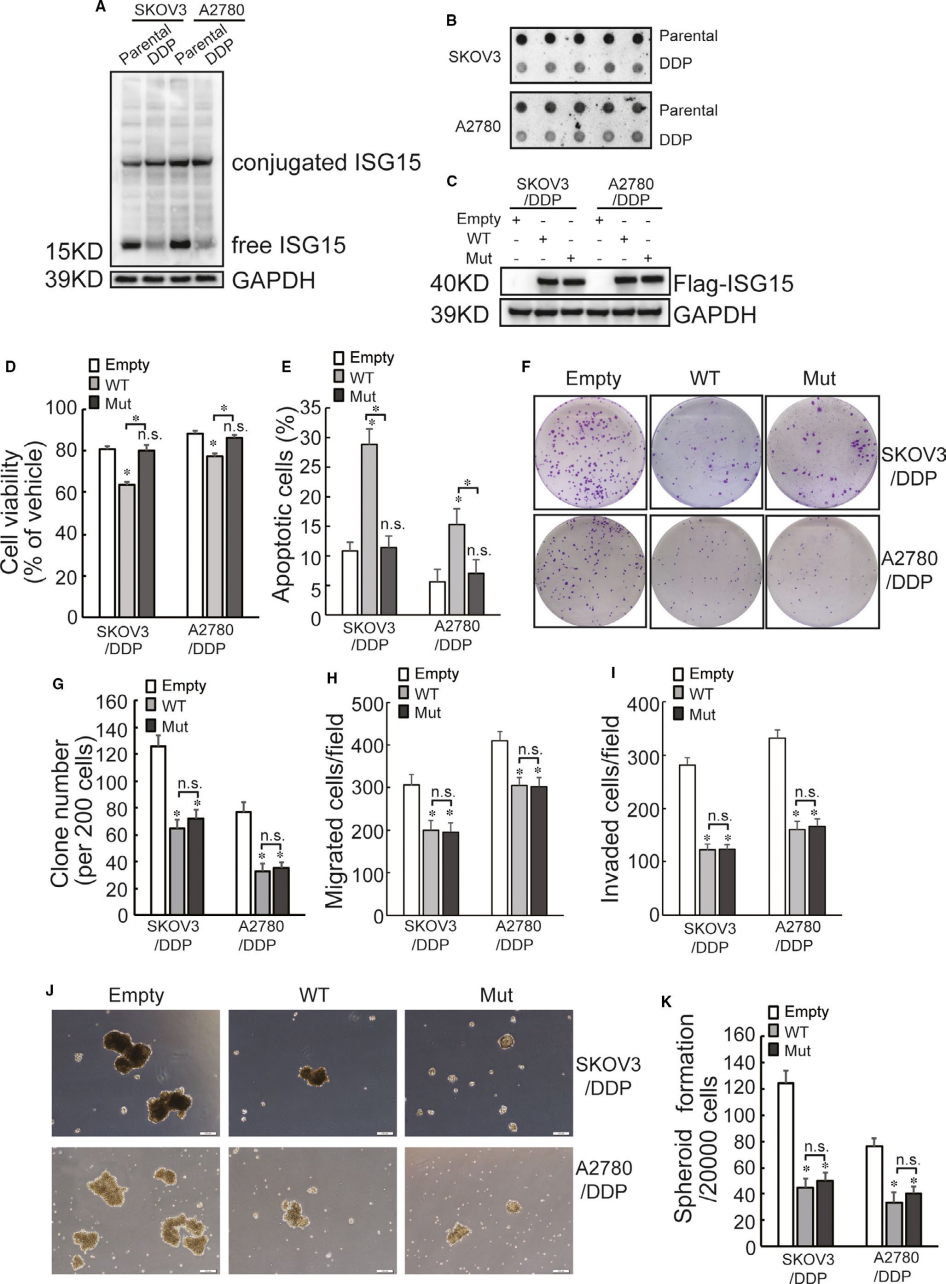
Downregulated ISG15 expression promotes cancer stem cell like features of cisplatin‐resistant ovarian cancer cells. A, Total proteins were isolated from the indicated cells, ISG15 expression was evaluated using Western blot. B, Cells were cultured under serum‐free media and supernatant were collected after 3 d' culture, extracellular release of ISG15 was analysed using dot blot. C, Cells were infected with lentivirus containing wild type (WT) or nonconjugatable mutant (G156/157A) ISG15, ISG15 expression were confirmed by Western blot. D, The indicated cell were treated with 10 μg/mL of cisplatin for 24 h, and cell viability was assessed using CCK8 assay. E, The indicated cell were treated with 10 μg/mL of cisplatin for 24 h, cell apoptosis was analysed. F‐K, capacities of colony formation (F‐G), migration (H), invasion (I) and spheroid formation (J‐K) were evaluated in the indicated cells. Representative images of colony formation (F) and spheroid formation (J) were presented. An asterisk (*) represents significant difference with *P* < .05. Error bars are indicative of means ± SD. n.s., not significant

ISG15 expression was explored in two pairs of cisplatin‐sensitive (SKOV3 and A2780) and cisplatin‐resistant (SKOV3/DDP and A2780/DDP) ovarian cancer cell lines. ISG15 expression in SKOV3/DDP and A2780/DDP cells was significantly lower than that in control partners (Figure 1A). As it was reported that ISG15 could be released to extracellular environment, ISG15 in culture supernatant was investigated using dot blot. Supernatant ISG15 also decreased in SKOV3/DDP and A2780/DDP cells (Figure 1B). These data indicated that ISG15 was downregulated in cisplatin‐resistant ovarian cancer cells. To study potential function of ISG15 downregulation in cisplatin‐resistant ovarian cancer cells, wild‐type (WT) or nonISGylatable mutant (G156/157A mutant, Mut) ISG15 was ectopically expressed in SKOV3/DDP and A2780/DDP cells using lentivirus (Figure 1C). It was shown that WT ISG15 decreased cell viability (Figure 1D) and increased cell apoptosis (Figure 1E) of SKOV3/DDP and A2780/DDP cells exposed to 10 μg/mL of cisplatin, while Mut ISG15 exerted no obvious effects. Cancer stem cell‐like features including capacities of colony formation, migration and invasion, as well as spheroid formation, were also evaluated. Both WT and Mut ISG15 significantly decreased colony formation (Figure 1F,G), migration (Figure 1H), invasion (Figure 1I) and spheroid formation (Figure 1J,K) of SKOV3/DDP and A2780/DDP cells.

### 3.2 *ISG15* knock‐down promotes cancer stem cell‐like features in cisplatin‐sensitive ovarian cancer cells

**FIGURE 2:**
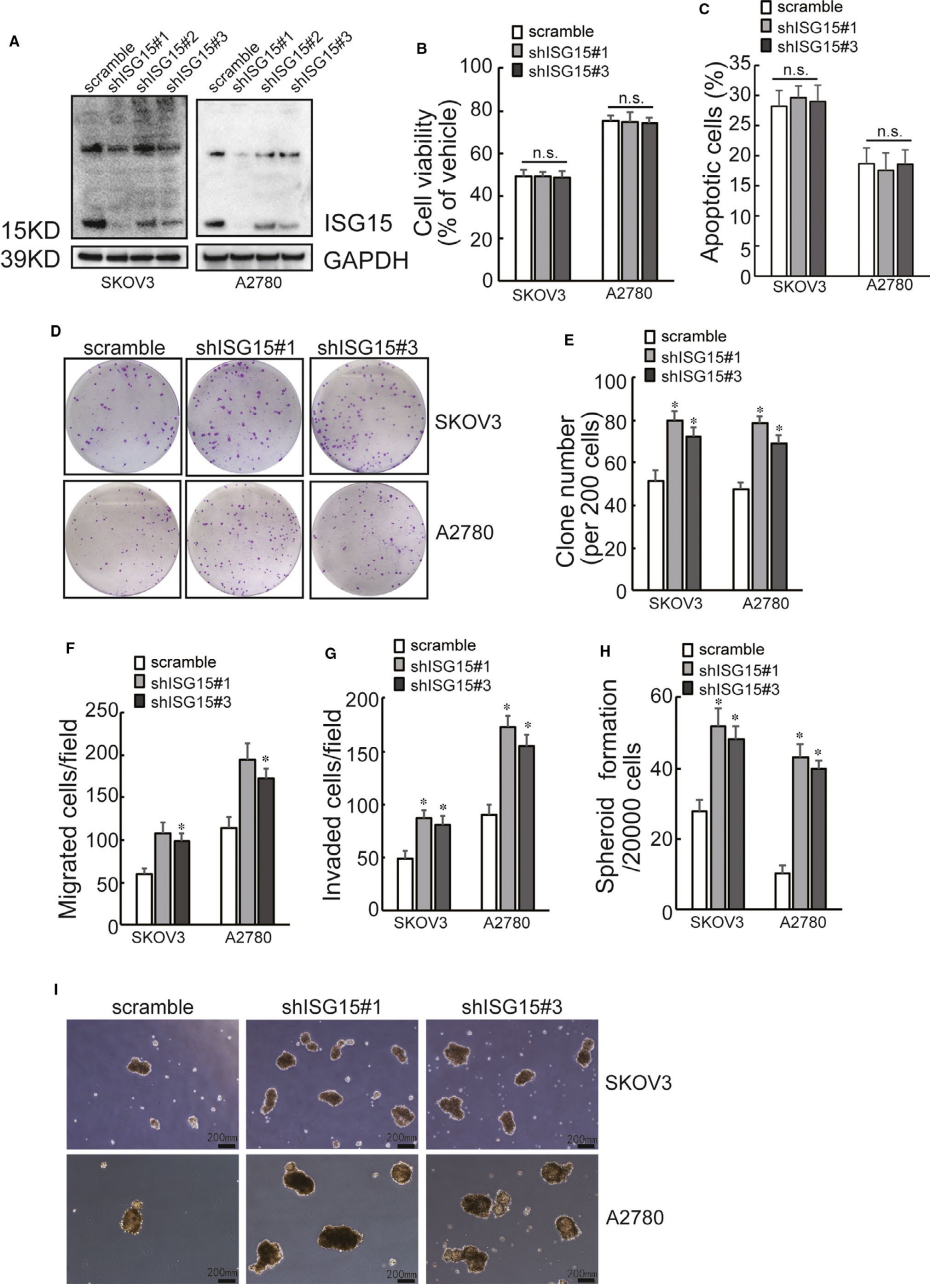
Loss of ISG15 rescues cancer stem cell like phenotypes of cisplatin‐sensitive ovarian cancer cells. A, SKOV3 and A2780 cells were infected with lentivirus containing scramble or shISG15, downregulation of ISG15 was confirmed by Western blot. B, The indicated cell viability was assessed using CCK8 assay, after exposure to 10 μg/mL of cisplatin for 24 h. C, The indicated cell were treated with 10 μg/mL of cisplatin for 24 h, cell apoptosis was analysed. D‐I, capacities of colony formation (D‐E), migration (F), invasion (G) and spheroid formation (H‐I) were analysed in the indicated cells followed by the infection with lentivirus containing scramble or shISG15. Representative images of colony formation (D) and spheroid formation (I) were presented. An asterisk (*) represents significant difference with *P* < .05. Error bars are indicative of means ± SD. n.s., not significant

ISG15 expression was knock‐down using lentivirus containing shRNAs specific against *ISG15* (shISG15). Two shRNAs (shISG15#1 and shISG15#3) exhibited potent suppression of *ISG15* expression in both SKOV3 and A2780 cells (Figure 2A). It was demonstrated that *ISG15* downregulation produced no obvious effect on SKOV3 or A2780 cell viability (Figure 2B) and cell apoptosis (Figure 2C) in the presence of cisplatin. However, ISG15 knock‐down significantly increased cancer stem cell‐like features of SKOV3 and A2780 cells, including colony formation (Figure 2D,E), migration (Figure 2F), invasion (Figure 2G) and spheroid formation (Figure 2H,I).

### 3.3 Ectopic ISG15 expression suppresses tumour formation in nude mice and low ISG15 expression is associated with poor prognosis in patients with ovarian cancer

**FIGURE 3:**
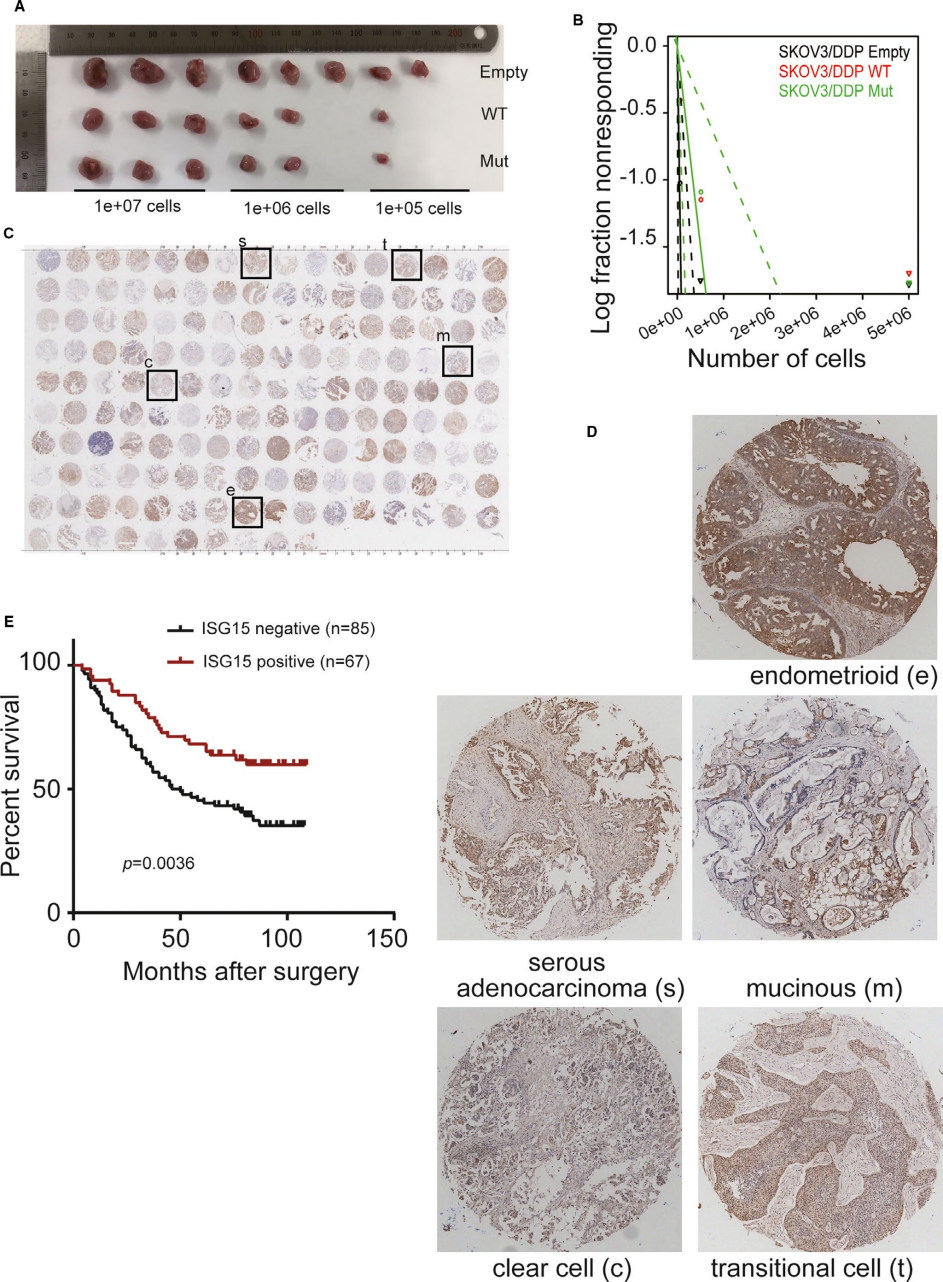
Ectopic ISG15 expression suppresses tumour formation in nude mice and low ISG15 expression is associated with poor prognosis. A, Serially diluted cells were inoculated intracutaneously into nude mice. Experimental mice were killed and tumours were excised on day 28 (n = 3 mice/group). B, ELDA was used to predict the frequency of cancer stem cell, and the limiting dilution model was plotted as log‐fraction. The log‐active cell fraction was presented as the dotted lines and the 95% confidence interval was also given. *P < .05. NS not significant. C, Immunohistochemistry staining with ISG15 using epithelial ovarian cancer tissue microarray. D, Representative immunohistochemistry staining indicated in (C). E, ISG15 expression was categorized as high and low expression, and Kaplan‐Meier plot was used to analyse the overall survival of patients with ovarian cancer. Log‐rank test was used to determine *P*‐value

Tumour formation in vivo was then investigated in nude mice. Results exhibited that both WT and Mut ISG15 suppressed the growth of SKOV3/DDP xenografted tumours in nude mice (Figure 3A). Online limiting dilution analysis using ELDA (http://bioinf.wehi.edu.au/software/elda/) showed that the frequency of repopulating SKOV3/DDP cells with WT or Mut ISG15 was 1/93310, while the frequency of repopulating control cells was estimated as 1/10629 (Figure 3B, *P* = .0457, control vs WT or Mut ISG15).

ISG15 expression was also analysed by immunohistochemical staining in different epithelial ovarian cancer tissue microarray, including serous adenocarcinoma, mucinous carcinoma, endometrial carcinoma, clear cell carcinoma and transitional cell carcinoma (Figure 3C,D). Based on the expression of ISG15 in 152 clinical samples, we divided the samples into negative (n = 85) and positive (n = 67) groups to investigate the relationship between clinical features and ISG15 expression (Table 1). According to chi‐squared tests, low ISG15 expression was highly associated with histological grade, TNM classification and death rate (with *P* < .01, Table 1). Survival data analysis revealed that negative ISG15 expression was associated with significantly shortened survival time (*P* = .0036) after removal of the primary tumour (Figure 3E). With respect to overall survival, the Cox proportional hazards model demonstrated that ISG15 was not an independent prognostic factor (hazard ratio = 1.922 (95% confidence interval, 1.239‐2.982), *P* = .5202).

**TABLE 1:** Relationship between ISG15 expression and clinical characteristics in ovarian cancer

Clinical characteristics	ISG15 expression (N0)		*P*‐value
	Negative	Positive	
Histologic grade			
G1	2	6	.000
G2	12	23	
G3	43	33	
G4	28	5	
Stage			
I	8	6	.715
II	3	1	
III	38	32	
IV	36	28	
T classification			
T1	2	6	.001
T2	12	23	
T3	71	38	
N classification			
N0	52	58	.001
N1	33	9	
M classification			
M0	57	62	.000
M1	28	5	
Survival status			
Death	55	26	.004
Survival	30	41	

### 3.4 Nascent *ISG15* mRNA decreased and degraded *ISG15* mRNA increased in cisplatin‐resistant ovarian cancer cells

**FIGURE 4:**
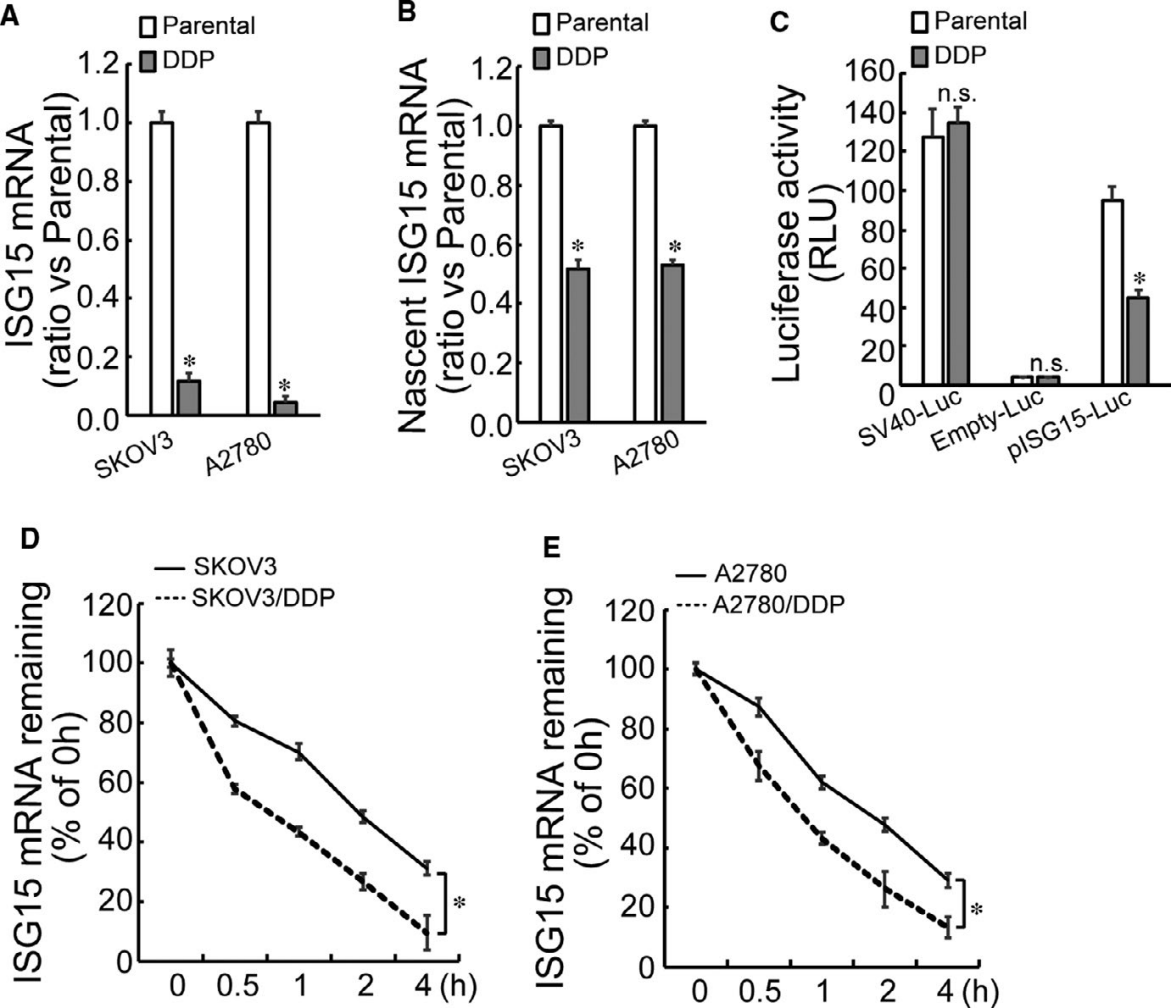
Transcriptional regulation of *ISG15* and stability of *ISG15* mRNA. A, Total RNA was isolated from the indicated cells, and *ISG15* mRNA expression was analysed by real‐time RT‐PCR. B, Newly synthesized *ISG15* mRNA was captured using a ClickiT in the indicated cells and then measured using real‐time RT‐PCR. C, The luciferase reporter vector bearing SV40, null (Empty) or *ISG15* promoter (pISG15) was constructed. SKOV3 and SKOV3/DDP cells were cotransfected with the indicated luciferase reporter vector and Renilla reporter vector. The activity of luciferase and Renilla was analysed 2 d later. Renilla activity was used to normalize the luciferase activity. D‐E, Cisplatin‐sensitive and cisplatin‐resistant SKOV3 (D) or A2780 cells (E) were exposed to actinomycin D for the indicated time, and *ISG15* mRNA was measured using real‐time RT‐PCR *ISG15* mRNA levels were normalized to 18S rRNA and plotted as a percentage from three experiments repeated independently. An asterisk (*) represents significant difference with *P* < .05. Error bars are indicative of means ± SD. n.s., not significant

The obvious involvement of ISG15 in the cancer stem cell‐like features urged us to elucidate the mechanisms underlying regulation of *ISG15* expression. Compared with the cisplatin‐sensitive SKOV3 and A2780 cells, *ISG15* mRNA decreased by more than 80% in SKOV3/DDP and A2780/DDP cells (Figure 4A). On the other hand, only about 40% reduction of nascent *ISG15* mRNA was detected (Figure 4B). These data indicated that both transcriptional and post‐transcriptional factors might be responsible for ISG15 downregulation in ovarian cancer cells. Firstly, to confirm the potential transcriptional suppression of ISG15 in cisplatin‐resistant ovarian cancer cells, the potential *ISG15* promoter was cloned into a promoter‐free luciferase reporter construct. SKOV3 cells and its cisplatin‐resistant partner SKOV3/DDP cells were transfected with SV40‐Luc (positive control), empty‐Luc (negative control) or pISG15‐Luc construct, and luciferase activity was analysed. Significant decrease in the luciferase activity of the reporter construct with *ISG15* promoter (pISG15‐luc construct) was observed in SKOV3/DDP cells (Figure 4C), inferring that *ISG15* promoter recruited negative regulators in cisplatin‐resistant ovarian cancer cells. Subsequently, degradation rate of *ISG15* mRNA was also accessed. The stability of *ISG15* mRNA was lower in SKOV3/DDP and A2780/DDP than in SKOV3 and A2780 cells (Figure 4D,E). Together, *ISG15* mRNA decreased at both transcriptional activation and post‐transcriptional stability in cisplatin‐resistant ovarian cancer cells.

### 3.5 *ISG15* is downregulated by KLF12 at the transcriptional activation level *via* the CACCC elements located in −1187/−1013 and −672/−503 fragment

**FIGURE 5:**
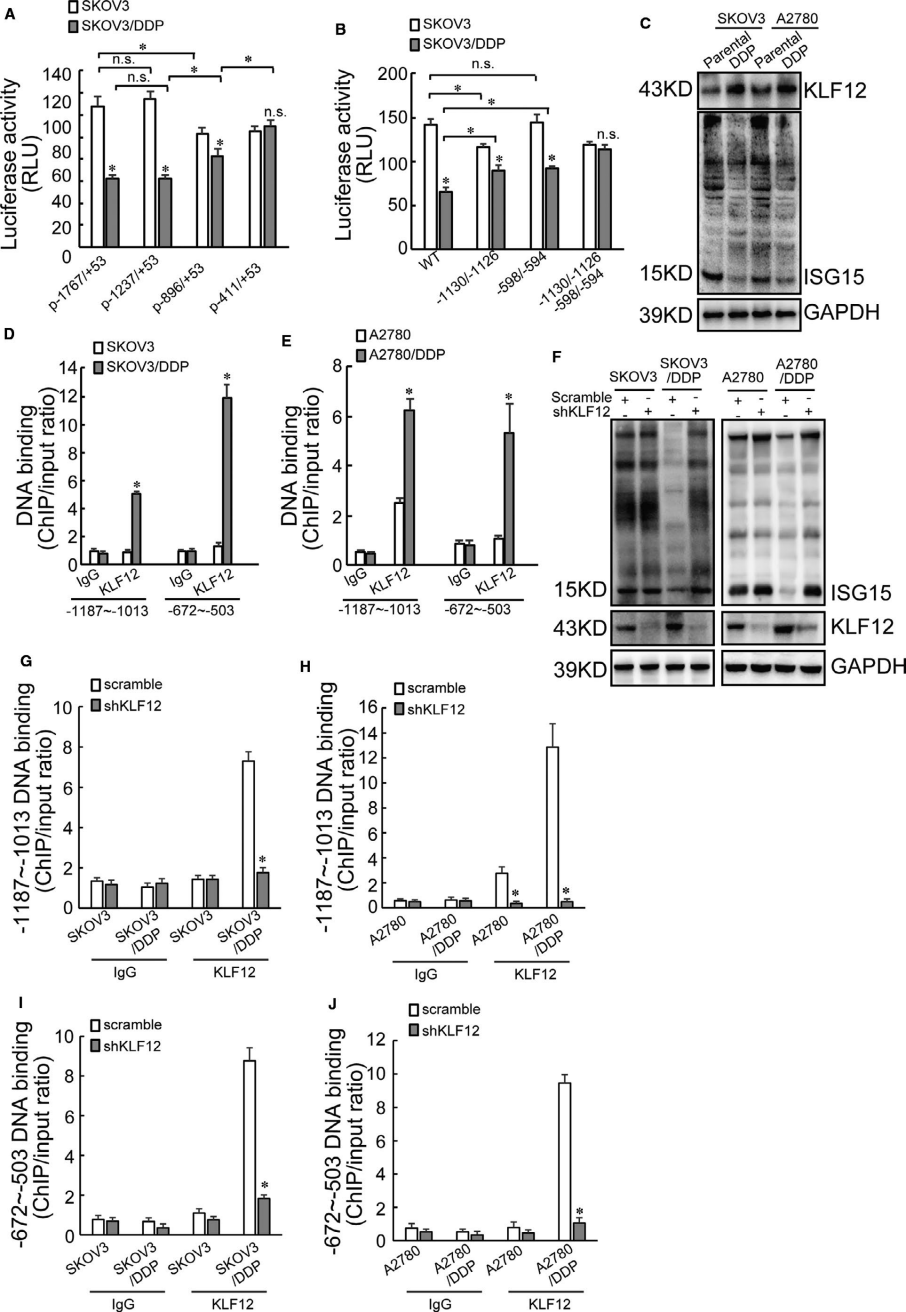
*ISG15* transcription is regulated by KLF12 *via* the CACCC element located in −1187/−1013 and −672/−503 fragment. A, The luciferase reporter vector bearing p‐1767/+53, p‐1237/+53, p‐896/+53 or p‐411/+53 segment of *ISG15* promoter was constructed. The indicated luciferase reporter vector and Renilla reporter vector was used to transfect SKOV3 or SKOV3/DDP cells. Luciferase activity was evaluated 2 d later. Renilla activity was used to normalize luciferase activity. B, Two potential binding motifs in *ISG15* promoter for KLF12 (CACCC elements) were located at −1130/−1126 and −598/−594 sequence. Luciferase reporter bearing *ISG15* promoter with −1130/−1126 deletion, −598/−594 deletion or both of them deletion was constructed. SKOV3 or SKOV3/DDP cells were cotransfected with the indicated luciferase reporter vector and Renilla reporter vector. Luciferase activity was evaluated 2 d later. Renilla activity was used to normalize luciferase activity. C, Total proteins were derived from paired cisplatin‐sensitive and cisplatin‐resistant SKOV3 or A2780 cells, and KLF12 and ISG15 expression was assessed using Western blot. D‐E, ChIP assay of KLF12 recruitment to the indicated DNA fragment of *ISG15* promoter in cisplatin‐sensitive and cisplatin‐resistant SKOV3 (D) and A2780 (E) cells. F, Cisplatin‐sensitive and cisplatin‐resistant SKOV3 or A2780 cells were infected with lentivirus containing scramble or shKLF12, ISG15 and KLF12 expression was analysed using Western blot. G‐H, ChIP assay of KLF12 recruitment to the −1187/−1013 fragment of *ISG15* promoter in cisplatin‐sensitive and cisplatin‐resistant SKOV3 or A2780 cells transfected with scramble or shKLF12. I‐J, ChIP assay of KLF12 recruitment to the −672/−503 fragment of *ISG15* promoter in cisplatin‐sensitive and cisplatin‐resistant SKOV3 or A2780 cells transfected with scramble or shKLF12. An asterisk (*) represents significant difference with *P* < .05. Error bars are indicative of means ± SD. n.s., not significant

To identify the potential cis‐acting regulatory elements located on the ISG15 promoter, luciferase reporter constructs containing truncated ISG15 promoter were constructed. Luciferase activity demonstrated that reporter construct containing −411/+53 segment did not generate any difference in SKOV3 and SKOV3/DDP cells (Figure 5A), excluding existence of any regulatory element spanning this region. Constructs containing −1767/+53 and −1237/+53 segments demonstrated similar suppressive effect on the luciferase activities (Figure 5A), indicating that no regulatory element between −1767 and −1238 region responsible for suppressing ISG15 expression in cisplatin‐resistant ovarian cancer cells. Moreover, p‐896/+53 was significantly less effective than p‐1767/+53 and p‐1237/+53, respectively (Figure 5A). Hence, it was inferred that there were regulatory elements spanning −1236/−897 and −896/−412 region, which might be responsible for suppressing ISG15 expression in cisplatin‐resistant ovarian cancer cells. Furthermore, there might be some regulatory element located on −1237/−897 region responsible for transcriptional ISG15 activation in SKOV3 cells. CACCC element is potential binding motifs for KLFs to regulate gene expression.^35^ Two CACCC elements were located at −1130/−1126 and −598/−594 sequences of ISG15 promoter. Luciferase reporter constructs bearing *ISG15* promoter with deletion of −1130/−1126, −598/−594, or both of them were then constructed. Luciferase activity of the reporter construct with wild‐type promoter −1130/−1126 deletion or −598/−594 deletion significantly decreased in SKOV3/DDP cells than in SKOV3 cells respectively (Figure 5B). Compared with the reporter construct bearing wild‐type promoter, luciferase activity of the reporter construct with −1130/−1126 deletion or −598/−594 deletion was significantly increased in SKOV3/DDP cells (Figure 5B). Luciferase activity of the reporter construct bearing both −1130/−1126 and −598/−594 deletion had no difference in SKOV3/DDP cells and SKOV3 cells (Figure 5B). Thereby, these data indicated that CACCC element located in −1130/−1126 and −598/−594 region of *ISG15* promoter was transcriptional repressor of the *ISG15* in SKOV3/DDP cells.

Coexpression of ISG15 and KLF family members was analysed in ovarian cancer using online data (starBase v3.0 project). Obvious negative correlation of ISG15 was observed with KLF12 (*R* = −0.287, *P* = 1.25e−8) in 379 ovarian cancers (Table S1). Pan‐cancer analysis using starBase v3.0 project observed negative correlation between ISG15 and KLF12 in multiple cancers (Table S2). Western blot also demonstrated that KLF12 increased in cisplatin‐resistant ovarian cancer cells (Figure 5C). To further demonstrate the molecular mechanisms regulated by KLF12, ChIP‐PCR assay was then performed. Compared with cisplatin‐sensitive parental cells, recruitment of KLF12 by both −1187/−1013 and −672/−503 fragments was consistently increased in SKOV3/DDP (Figure 5D) and A2780/DDP cells (Figure 5E). KLF12 was then knocked down using lentivirus containing shKLF12 in cisplatin‐sensitive and cisplatin‐resistant SKOV3 or A2780 cells. ISG15 increased in SKOV3/DDP and A2780/DDP cells with KLF12 knock‐down (Figure 5F). ChIP‐PCR data showed that KLF12 knock‐down had no effect on the recruitment of KLF12 to −1187/−1013 fragment of *ISG15* promoter in SKOV3 and A2780 cells (Figure 5G), while KLF12 recruitment was significantly decreased due to KLF12 knock‐down in SKOV3/DDP and A2780/DDP cells (Figure 5H). The identical effects have arisen on −672/−503 fragment from KLF12 knock‐down (Figure 5I,J).

## 4 DISCUSSION

Cisplatin is utilized as the first‐line medicine for patients with various cancers including ovarian cancer. Although the survival length of patients was largely improved by the combination of cisplatin chemotherapy and cytoreduction, its therapeutic application in ovarian cancer was compromised by the drug resistance and adverse side‐effect. Therefore, understanding the molecular mechanisms underlying cisplatin resistance might lead to potential therapeutic strategy for ovarian cancer treatment.

ISG15 plays an apparently contradictory role in cancers. On one side, ISG15 is highly expressed and functions as a tumour‐promoting molecule in some cancers,^7^, ^10^, ^36^, ^37^ and its high expression contributes to cancer progression, including oesophageal,^38^ oral,^39^ nasopharyngeal^11^ and pancreatic cancer.^7^ It has been reported that free ISG15 promotes cancer stem cell‐like phenotypes of PDAC *via* autocrine^40^ and paracrine‐mediated pattern.^7^ On the other side, ISG15 and ISG15 conjugated targets are also been reported to suppress progression of some cancers, such as lung cancer,^13^, ^41^ glioblastoma^42^ and cervical cancer.^14^ ISG15 upregulation has been reported to promote cancer stem cell phenotype and increases cell resistance to cisplatin (DDP) treatment in nasopharyngeal carcinoma,^11^ while *ISG15* downregulation increases cisplatin resistance in lung cancer.^43^ Consistent with the phenomena observed in lung cancer, we currently reported that significant decrease in ISG15 was observed in cisplatin‐resistant ovarian cancer cell lines. Moreover, ectopic overexpression of wild‐type ISG15 increased the sensitivity of cisplatin‐resistant cell lines. Interestingly, paradoxical role of ISG15 has been assigned to breast cancer, as free ISG15 plays a antitumour role by activating immune system in vivo in breast cancer,^15^ while the conjugated ISG15 triggers a malignant transformation of breast cells.^12^, ^44^ Thereby, the difference between free and conjugated form of ISG15 might be an alternative explanation for its paradoxical function in distinct cancers. Our work showed that only conjugatable ISG15 (wild type) increased the sensitivity of cisplatin in ovarian cancer cells, while both conjugatable and nonISGylatable mutant ISG15 were involved in CSC‐like characters. In addition, knock‐down of ISG15 did not alter the responsiveness to cisplatin, but promoted CSC‐like features of sensitive ovarian cancer cells. Importantly, our data showed that ISG15 positive expression was correlated with good prognosis in the patients with ovarian cancer. These data indicated that only ISG15 downregulation may not be enough to induce cisplatin resistance, but its downregulation might be implicated in maintenance of CSC‐like features and make a significant contribution to cisplatin resistance of ovarian cancer cells.

ISG15 expression was suppressed at transcriptional level, as well as post‐transcriptional levels in cisplatin‐resistant ovarian cancer cells. We focused on the study at the transcriptional level and demonstrated that KLF12 repressed the transcriptional activation of ISG15. Functionally, KLF1 and KLF5 act as transcriptional activators, while KLF 12 serves as transcriptional repressor by interacting with carboxy‐terminal binding protein (CtBP).^45^ KLF9 is reported to be recruited to the *ISG15* promoter region and prevents colorectal cancer by repression of *ISG15*.^46^ In the present study, we disclosed that KLF12 was upregulated and recruited by *ISG15* in cisplatin‐resistant ovarian cancer cells. Moreover, KLF12 knock‐down significantly increased the expression of *ISG15* in cisplatin‐resistant SKOV3 cells. Therefore, we inferred that *ISG15* was downregulated by KLF12 *via* binding with the CACCC element in cisplatin‐resistant ovarian cancer cells, implicated in maintenance of CSC‐like features. To our best knowledge, for the first time, the current study elaborated that attenuated ISG15 maintains CSC features of ovarian cancer cells, which was involved in cisplatin resistance. Furthermore, KLF12 plays an important role to decrease *ISG15* expression in cisplatin‐resistant SKOV3 cells. Therefore, these studies might suggest that KLF12 might function as a potential target to increase *ISG15* expression for inhibition of CSC phenotype in the treatment of cisplatin‐resistant ovarian cancer.

## CONFLICT OF INTEREST

The authors declare no conflict of interest.

## AUTHOR CONTRIBUTIONS

**Jing Yan:** Data curation (equal); funding acquisition (equal); writing‐original draft (lead). **Qi Zhang:** Formal analysis (lead); methodology (equal); software (lead). **Jiamei Wang:** Investigation (equal); methodology (equal). **Huaiyu Qiao:** Methodology (equal). **Lingyue Huyan:** Methodology (supporting). **Baoqin Liu:** Data curation (equal); funding acquisition (supporting). **Chao Li:** Funding acquisition (supporting). **Jingyi Jiang:** Methodology (supporting). **Fuying Zhao:** Methodology (supporting). **Huaqin Wang:** Data curation (equal); investigation (equal); project administration (equal).

## Supporting information

Table S1‐2

## Data Availability

The data that support the findings of this study are available from the corresponding author upon reasonable request.
