# Supplementary material for: ISG15 is downregulated by KLF12 and implicated in maintenance of cancer stem cell‐like features in cisplatin‐resistant ovarian cancer
Source: J Cell Mol Med. 2021 Apr 2;25(9):4395–407. doi: 10.1111/jcmm.16503 (PMC8093991; doi:10.1111/jcmm.16503)
Supplement: Supplementary file 1 — Table S1‐2 [file JCMM-25-4395-s001.pdf]

Table S1

Co-expression of ISG15 and KLF family members in ovarian cancer (n=379)

KLF family	coefficient-R	p-value
KLF1	0.232	5.16e-6
KLF3	-0.149	3.60e-3
KLF5	-0.266	1.52e-7
KLF6	0.097	6.00e-2
KLF9	0.128	1.27e-2
KLF11	-0.147	4.05e-3
KLF12	-0.287	1.25e-8
KLF13	-0.125	1.47e-2
KLF15	-0.113	2.75e-2

Data Source: starBase v3.0 project

Table S2

Co-expression of Pan-Cancer Analysis for ISG15 and KLF12

Cancer	Smaple Number	coefficient-R	p-value
Breast Invasive Carcinoma	1104	-0.380	2.38e-39
Cervical Squamous Cell Carcinoma and Endocervical Adenocarcinoma	306	-0.153	7.44e-3
Colon Adenocarcinoma	471	-0.225	7.64e-7
Lymphoid Neoplasm Diffuse Large B-cell Lymphoma	48	-0.382	7.32e-3
Head and Neck Squamous Cell Carcinoma	502	-0.481	2.08e-30
Kidney Chromophobe	65	-0.349	4.36e-3
Kidney Renal Clear Cell Carcinoma	535	-0.320	3.65e-14
Kedney Renal Papillary Cell Carcinoma	289	-0.565	9.71e-26
Brain Lower Grade Glioma	529	-0.383	6.11e-20
Liver Hepatocellular Carcinoma	374	-0.223	1.39e-5
Lung Adenocarcinoma	526	-0.278	8.65e-11
Mesothelioma	86	-0.386	2.41e-4
Ovarian Serous Cystadenocarcinoma	379	-0.287	1.25e-8
Pheochromocytoma and Paraganglioma	183	-0.197	7.65e-3
Rectum Adenocarcinoma	167	-0.216	5.02e-3
Sarcoma	263	-0.213	5.21e-4
Skin Cutaneous Melanoma	471	-0.096	3.75e-2
Stomach Adenocarcinoma	375	-0.177	5.92e-4
Testicular Germ Cell Tumors	156	-0.312	7.51e-5
Thymoma	119	-0.573	9.36e-12
Uterine Corpus Endometrial Carcinoma	548	-0.146	5.90e-4

Data Source: starBase v3.0 project
